# Effects of organic trace minerals chelated with oligosaccharides on growth performance, blood parameters, slaughter performance and meat quality in sheep

**DOI:** 10.3389/fvets.2024.1366314

**Published:** 2024-03-21

**Authors:** Runze Zhang, Manlin Wei, Jianqun Zhou, Zaibin Yang, Ming Xiao, Liu Du, Meili Bao, Ji Ju, Chenyang Dong, Yongjie Zheng, Hailin Bao

**Affiliations:** ^1^College of Animal Science and Technology, Inner Mongolia Minzu University, Tongliao, China; ^2^Nanning Zeweier Feed Limited Liability Company, Nanning, China; ^3^College of Animal Science and Veterinary Medicine, Shandong Agricultural University, Tai'an, Shandong, China; ^4^Horqin Left Wing Rear Banner National Vocational and Technical School, Tongliao, China

**Keywords:** oligosaccharides chelated organic trace minerals, digestive enzyme activity, growth performance, blood parameters, slaughter performance, amino acids in sheep meat

## Abstract

The present study assessed the effects of oligosaccharide-chelated organic trace minerals (OTM) on the growth performance, digestive enzyme activity, blood parameters, slaughter performance, and meat quality indexes of mutton sheep. A total of 60 East Ujumuqin × small-tailed Han crossbred mutton sheep were assigned to two groups (10 duplicates per group) by body weight (26.12 ± 3.22 kg) according to a completely randomized design. Compared to the CON group, the results of the OTM group showed: (1) no significant changes in the initial body weight, final body weight, dry matter intake, average daily gain, and feed conversion ratio (*p* > 0.05); (2) the activities of trypsin, lipase, and amylase in the jejunum were significantly increased (*p* < 0.05); (3) serum total protein, albumin, and globulin of the blood were significantly increased (*p* < 0.05), and the growth factor interleukin IL-10 was significantly higher (*p* < 0.05), while IL-2, IL-6, and γ-interferon were significantly lower (p < 0.05). Immunoglobulins A, M, and G were significantly higher (*p* < 0.05); (4) the live weight before slaughter, carcass weights, dressing percentage, eye muscle areas, and GR values did not differ significantly (*p* > 0.05); (5) shear force of mutton was significantly lower (*p* < 0.05), while the pH_45min_, pH_24h_, drip loss, and cooking loss did not show a significant difference (*p* > 0.05). The content of crude protein was significantly higher (*p* < 0.05), while the ether extract content was significantly reduced (*p* < 0.05), but no significant difference was detected between moisture and ash content; (6) the total amino acids, essential amino acids, semi-essential amino acids, and umami amino acids were significantly increased (*p* < 0.05). Although umami amino acids were not significant, the total volume increased (*p* > 0.05). Among these, the essential amino acids, threonine, valine, leucine, lysine in essential amino acids and arginine were significantly increased (*p* < 0.05). Also, non-essential amino acids, glycine, serine, proline, tyrosine, cysteine, and aspartic acid, were significantly higher (*p* < 0.05). The content of alanine, aspartate, glutamic acid, phenylalanine, and tyrosine in umami amino acids was significantly higher (*p* < 0.05).

## 1 Introduction

Sheep are traditionally reared by grazing, but with improved raising of mutton sheep and people’s attention to the safety of livestock products, farm feeding is gaining popularity. The availability of feed nutrients is one of the major issues in captive animal production, and feeding only native forage lacks the essential minerals needed to maintain optimal animal performance (1). In this case, supplementation with trace minerals is necessary to maximize animal performance. Trace minerals, such as copper (Cu), zinc (Zn), manganese (Mn), cobalt (Co), selenium (Se), and iodine (I), are essential for animals (2). Although these minerals constitute <0.01%, they are essential in maintaining normal physiological activities, such as hematopoiesis, immune response, energy metabolism, enzyme activity, and reproductive functions, in an animal’s body (3, 4). In addition, most trace minerals are involved in the composition of oxidoreductases and other enzymes that participate in the redox reactions or bind to oxygen radicals to alleviate oxidative stress in the body. Trace mineral deficiencies in livestock are known to negatively affect various biological functions, growth performance, and health status of the animals (5, 6). Therefore, trace mineral supplements are usually added to the water and feed for animals or supplemented through oral formulations, rumen instillation, or direct injection (7). The most common method used in livestock production is to supplement feed with trace inorganic salts, such as sulfates, oxides, carbonates, and chlorides. However, in ruminants, the ionic bonds in inorganic salts dissociate as they flow through the digestive tract, interacting with other components of the gut to form complexes and prevent absorption, thus reducing the bioavailability and absorption efficiency of trace minerals (8, 9). To ensure adequate intake, trace element minerals are usually over-added to feed, which increases the residual components, resulting in accumulation and toxicity in the animal’s body. In addition, the excretion of excess inorganic salts through feces and urine adds to the environmental burden (10). Conversely, organic trace minerals can avoid the adverse effects of interfering components and gastric acid in the digestive tract of animals, such that the metal ions can reach the absorption site, improving the absorption and utilization of trace mineral elements, such as Cu, Zn, Fe, and Mn, and reducing the environmental pollution and wastage of resources (11).

Oligosaccharides can promote the growth performance of animals, improve the immune capacity improve meat quality and other effects (12–14). In recent years, oligosaccharides amino acids and other advanced chelating technology in mineral additives have been widely used, chelating organic trace minerals because of its small antagonism in the stomach and other dietary components, can improve immunity, promote productivity and affect meat quality and other effects, has been confirmed in many tests (15–17).

Oligosaccharide-chelated organic trace minerals (OTMs) are the products of a new chelating technology developed in recent years. It is composed of molasses and trace minerals, such as Fe, Cu, Mn, Zn, Co, I, and Se. The OTMs are lipophilic and do not need ligands to enter the animal’s body as they can be directly absorbed by the animal through multiple channels of glycolipids, glycoproteins, and oligosaccharides on the cell surface. So, it is expected to have higher absorption and utilization efficiency and better production performance. Some studies have shown that OTMs are effective in pigs and poultry (18, 19), but only a few studies have been reported in ruminants. Therefore, the present study aimed to investigate the effects of OTM on growth performance, digestive enzyme activity, blood parameters, slaughter performance, and meat quality indexes of mutton sheep to provide an opportunity for OTM in ruminant production.

## 2 Materials and methods

### 2.1 Animal management, diets, and experimental design

The randomized design study was conducted at the Cooperative Experimental Animal Breeding Base of Inner Mongolia Minzu University (43.17-N,121.29-E, altitude 600 m) from November 2021 to February 2022. A total of 60 East Ujumuqin and small-tailed Han crossbreed mutton sheep aged 3–4 months were equally divided into a control group (CON) and experimental group (OTM) with three replicates in each group and 10 sheep in each replicate pen. The sheep in the two groups were fed control diets and test diets (Table 1) at 4:00 a.m. and 14:00 daily, respectively, with free drinking water and the same feeding and management methods. All the animals were vaccinated; therefore, the sheep were raised for 119 d, which included a preparation period of 14 d and a formal experimental period of 105 d. The basic diet of the mutton sheep of both groups was the same: inorganic trace minerals and oligosaccharides chelated OTMs with equal mineral content were added at 0.1% to the diets of both groups. The nutritional value of the feed was in line with the recommended NY/T816-2004 feeding standard for mutton sheep (Ministry of Agriculture of the People’s Republic of China, 2004). OTM products were provided by Nanning Zeweier Feed Co., Ltd. (Nanning, China).

**Table 1:** Composition and nutrient levels of the basal diets (dry matter basis)%.

Ingredients	Content, %	Nutrient levels	Content, %
Com	40.13	Dry matter, DM	66.75
Corn Silage	25.89	Ether extract, EE	5.77
soybean meal	6.73	Crude protein, CP	19.69
DDGS	6.54	Neutral detergent fiber, NDF	20.29
Peanut vine	4.78	Acid detergent fiber, ADF	13.85
Sunflower cake	4.29	Metabolic energy, ME(MJ/kg)	12.47
Weeds	3.94		
Soybean meal	2.68		
Alfalfa hay	1.45		
Bean stalk	1.44		
Limestone	0.67		
NaCl	0.36		
NaHCO_3_	1.00		
Premix①	0.10		
Total	100		

①The premix (containing Fe 45,000 mg/kg, Cu 10,000 mg/kg, Mn 20,000 mg/kg, Zn 45,000 mg/kg, Co 200 mg/kg, I 750 mg/kg, Se 250 mg/kg) consists of sulfate and sucrose complexes in CON and OTM groups, respectively. The digestibility energy is calculated value, and other nutrient contents are measured values.

### 2.2 Sample collection and measurement

At the beginning and end of the experiment, the mutton sheep were weighed on two consecutive days before morning feeding to calculate the first weight and the last weight. The feed consumption was measured once on three consecutive days of each month, and samples were withdrawn to determine the content of nutrients, including dry matter (DM), ether extract (EE), crude protein (CP), neutral detergent fiber (NDF), and acid detergent fiber (ADF) according to the AOAC (2005) method (20), as shown in Table 1. Also, dry matter intake (DMI), average daily gain (ADG), and feed conversion ratio (FCR) were calculated. At the end of the experiment, four mutton sheep in each replication were randomly selected for jugular vein blood sampling. The supernatant was collected by centrifugation of the blood samples at 5000 rpm for 15 min after standing for 2 h at room temperature and stored at −20°C for analysis. Six sheep in each group were randomly selected to determine the slaughter indexes, including carcass weight, pH_45min_, pH_24h_, eye muscle area, and GR value. About 1 kg of longissimus dorsi muscle of each sheep was sampled to assess the tenderness, cooking loss, dripping loss, and amino acids of the mutton. Approximately, 2 mL of jejunum chyme was collected to determine the enzyme activity.

### 2.3 Blood parameters

Total protein (TP), albumin (ALB), globulin (GLB), and urea in serum were analyzed on a fully automated hematology analyzer (Myriad BS-410, Shenzhen, China), and the level of globulin in serum was obtained by the following formula:

GLB=TP−ALB .

Trypsin, lipase, and amylase were detected using biochemical kits (Beijing Huaying Biological Biotechnology Institute, Beijing, China); serum levels of interleukin 2 (IL-2), interleukin 6 (IL-6), interleukin (IL-10), and γ-interferon (IFN-γ), immunoglobulins A (IgA), IgM, and IgG were measured using enzyme immunoassay kits (Beijing Huaying Biological Biotechnology Institute) on an enzyme labeling instrument (Huawei Delang DR-200BS, Wuxi, China) and A6 semi-automatic biochemistry instrument (Beijing Matsushige Technology, Beijing, China).

### 2.4 Measurement of slaughter parameters

At the end of the experiment, 12 sheep in each group were randomly selected to fast for 24 h and avoid water for 12 h. Then, the live weight before slaughter was recorded. After slaughter, the head, hooves, skin, and viscera were removed, the intestinal fat was stripped, the carcass weight was recorded, and the slaughter rate was calculated as follows:

$\begin{array}{l} \mathrm{Dressing}\;\mathrm{percentage}\;\left(\%\right)=\\ \;100\times \mathrm{carcass}\;\mathrm{weight}\;\left(\mathrm{kg}\right)/\mathrm{live}\;\mathrm{weight}\;\mathrm{before}\;\mathrm{slaughter}\;\left(\mathrm{kg}\right) \end{array}$ .

The longissimus dorsi muscle (between 12th to 13th ribs) was outlined with a tracing paper, and the eye muscle area was estimated using the following formula:

$\begin{array}{l} \mathrm{Eye}\;\mathrm{muscle}\;\mathrm{area}\;\left({\mathrm{cm}}^{2}\right)=\\ \;\mathrm{eye}\;\mathrm{muscle}\;\mathrm{width}\times \mathrm{eye}\;\mathrm{muscle}\;\mathrm{height}\times 0.7 \end{array}$ .

The pH, cooking loss, drip loss, and shear force of the longissimus dorsi muscle were determined according to the method described by Kou Yufei (21). The instrument used for pH is MATTHAUS pH-OPTO-STAR (Ludwigsburg, Germany). Shear force Measurement using instrument Muscle Tenderness Instrument C-LM38 (Beijing, China). The GR value refers to the fat content of the carcass and is measured by the thickness of the tissue between the 12^th^ and 13^th^ ribs at a distance of 11 cm from the spine. The content of moisture, CP, EE, and ash in mutton was determined according to the AOAC method (2005), and the organic matter was calculated as dry matter minus ash.

### 2.5 Determination of free amino acids of mutton

Sample pre-treatment: the longissimus dorsi muscle samples of each group were homogenized by mixing 200 mg meat sample with 1 mL ultra-pure water and swirling for 30 min before centrifugation (13,200 rpm, 4 min). Derivation process: 50 μL of the supernatant was mixed with 50 μL of protein precipitator (NVL, n-valine is used as an internal standard), followed by centrifugation (13,200 rpm, 4°C, 4 min). Subsequently, 8 μL of supernatant was mixed with 42 μL of borate buffer (pH 8.5) for instant separation. A volume of 20 μL derivative reagent (AQC amino acids and protein detection reagent) was added and the reaction was incubated at 55°C for 15 min. After cooling, 50 μL of the sample was withdrawn for evaluation. The instrument parameters were as follows: High-performance-liquid chromatography-four-pole ion TRAP tandem Mass Spectrometer HPLC-MS/MS (Shimadzu LC20AD-API 3200 MD TRAP) was carried out on MSL ab 45 + AA-C18 (150 × 4.6 mm diameter 5 um), column temperature: 50°C, mobile phase A organic phase: water (A-formic acid + B-heptafluorobutyric acid regulator) B aqueous phase: acetonitrile (A + B regulator). Ion source: +ESI Electrospray ion source IS:+5500 V (spray voltage) GS1: 55 psi (atomizing gas), GS 2: 60 psi (auxiliary gas). Scanning mode: MRM multi-reaction monitoring, CAD: Medium (impact gas), TEM: 500°C (atomization temperature) CUR: 20 psi (air screen gas), CXP: 2.0 (impact chamber exit voltage), EP: 10 (injection voltage). Amino acids kit (MSLAB-45 + AA batch No. MSLAB451561#) was purchased from Beijing Mass Spectrometry Medical Research Co. Ltd., methanol, and acetonitrile to detect 20 amino acids: tyrosine (Tyr), valine (Val), methionine (Met), isoleucine (Ile), leucine (Leu), phenylalanine (Phe), tryptophan (Trp), lysine (Lys), histidine (His), argnine (Arg), glutamine (Gln), glutamic acid (Glu), glycine (Gly), alanine (Ala), serine (Ser), proline (Pro), tyrosine (Tyr), cysteine (Cys), asparagine (Asn), and aspartic acid (Asp).

### 2.6 Statistical analysis

The experimental data were summarized using Microsoft Excel (2016). Student’s t-test was performed using SPSS 26.0 (IBM Co., Armonk, NY, United States). The data are expressed as means and standard errors, and *p* < 0.05 was considered a significant difference. The figure was developed using Microsoft Excel (2016).

## 3 Results

### 3.1 Growth performance

As shown in Table 2, adding OTM had no significant influence on the ADG, DMI, and FCR (*p* > 0.05).

**Table 2:** Effect of oligosaccharides chelated with organic trace minerals on growth performance of mutton sheep.

Items	CON	OTM	SEM	*p* value
Initial BW, kg	26.16	26.11	0.610	0.769
Final BW, kg	52.96	53.79	1.135	0.650
ADG, g/d	255.27	263.65	9.319	0.468
DMI, g/d	796.77	716.10	54.656	0.523
FCR	3.12	2.71	0.402	0.546

^1^CON, inorganic trace mineral group; OTM, oligosaccharides organic trace mineral group; same for all tables below; ADG, average daily gain; DMI, dry matter intake; FCR, feed conversion ratio; FCR = DMI/ADG.

### 3.2 Jejunal digestive enzyme parameters

**Figure 1:**
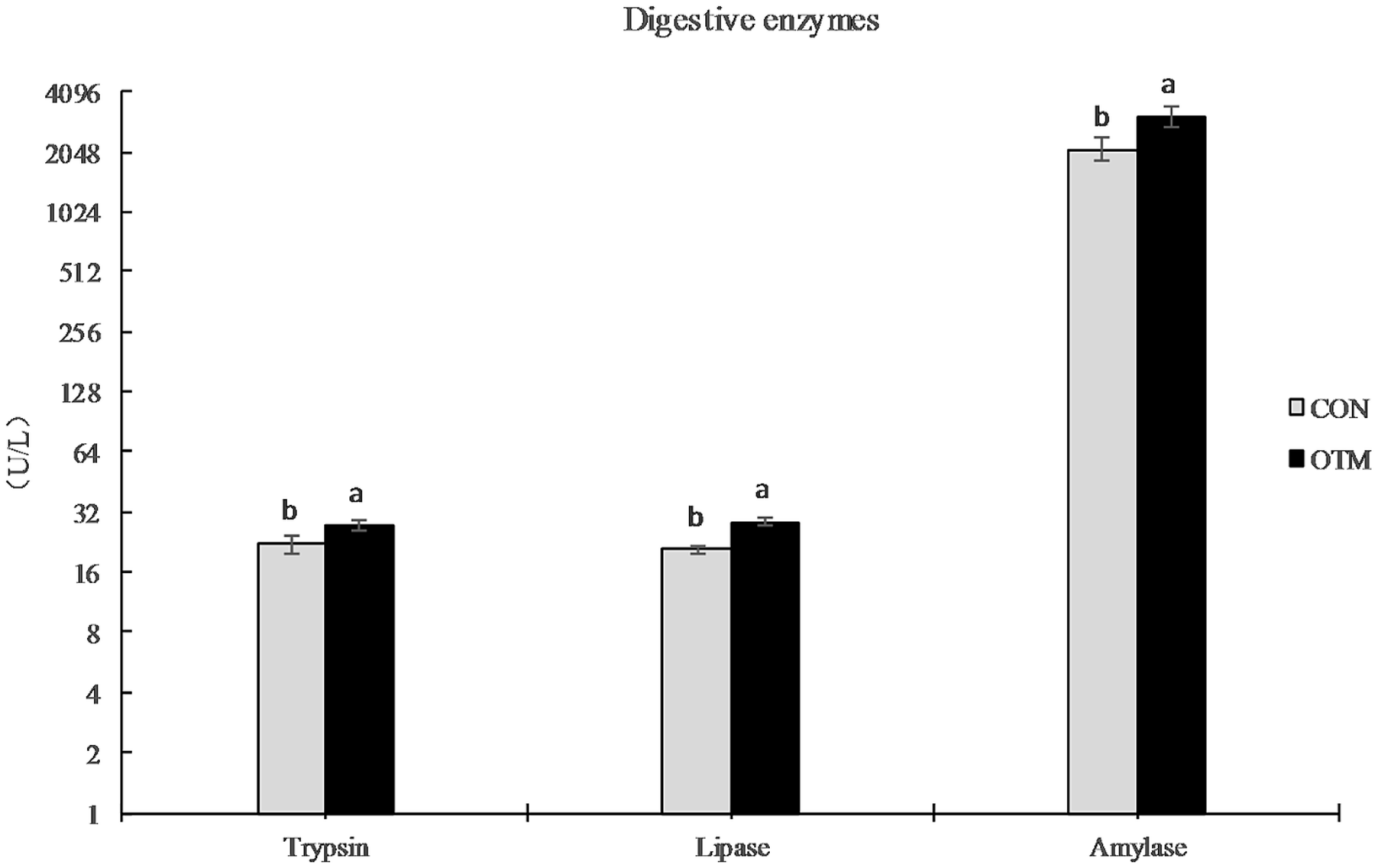
Effects of oligosaccharide chelated organic trace minerals on jejunal digestive enzymes in mutton sheep.

As shown in Figure 1, trypsin, lipase, and amylase activities in the jejunum chyme were significantly higher in the OTM group than in the CON group (*p* < 0.05).

### 3.3 Blood parameters

The blood parameters are shown in Table 3. The serum TP, ALB, and GLB were significant higher in the OTM group than in the CON group (*p* < 0.05), but urea did not differ significant between the two groups (*p* > 0.05). The IL-10 level was significantly higher in the OTM group (*p* < 0.05), but IL-2, IL-6, and IFN-γ levels were significantly lower in the OTM group (*p* < 0.05). Also, IgA, IgM, and IgG were significantly higher in the OTM than in the CON group (*p* < 0.05).

**Table 3:** Effects of oligosaccharides chelated with organic trace minerals on blood parameters of mutton sheep.

Items	CON	OTM	SEM	*p* value
TP, g/L	54.19^b^	67.07^a^	2.417	0.001
ALB, g/L	21.88^b^	27.80^a^	0.842	<0.001
GLB, g/L	32.30^b^	39.27^a^	1.849	0.009
UREA, mmol/L	6.27	6.91	0.390	0.470
IL-2, pg./mL	227.69^a^	179.24^b^	5.775	<0.001
IL-6, pg./mL	126.66^a^	112.07^b^	3.485	0.005
IL-10, pg./mL	15.14^b^	20.42^a^	0.528	<0.001
IFN-γ, pg./mL	32.59^a^	25.01^b^	1.086	<0.001
IgA, g/L	1.23^b^	1.45^a^	0.056	0.005
IgG, g/L	18.45^b^	20.06^a^	0.250	<0.001
IgM, g/L	1.09^b^	1.31^a^	0.068	0.020

^1^TP, total protein; ALB, albumin; GLB, globulin; IL-10, interleukin 10; IL-2, interleukin 2, IL-6, interleukin 6, IFN-γ, γ-interferon; IgA, immunoglobulins A; IgM, immunoglobulins M; IgG, immunoglobulins G. ^2^Data in the same row with different letters are significantly different (*p* < 0.05), whereas data without letters are not significantly different (*p* > 0.05), and the following tables are the same.

### 3.4 Slaughter performance and meat quality

As shown in Table 4, no significant difference was observed in the pre-slaughter live weight, carcass weight, dressing percentage, eye muscle area, and GR values between the two groups (*p* > 0.05). Table 5 shows that pH_45min,_ pH_24h_, shear force, cooking loss, and drip loss did not differ markedly between the two groups (*p* > 0.05). However, the CP of mutton was significantly higher and EE was significantly lower in the OTM group than in the CON group (*p* < 0.05), while the moisture content and organic matter of mutton did not differ significantly (*p* > 0.05). As shown in Table 6, Thr, Val, Leu, Lys, Arg, Asp, Gly, Ser, Pro, Tyr, and Cys content and the values of EAA, NEAA, and UAA were higher in the OTM group compared to the CON group (*p* < 0.05).

**Table 4:** Effect of oligosaccharides chelated with organic trace minerals on slaughter performance of mutton sheep.

Items	CON	OTM	SEM	*p* value
Live weight before slaughter, kg	55.13	53.54	2.126	0.604
Carcass weight, kg	25.38	24.36	1.608	0.508
Dressing percentage, %	46.05	45.54	0.886	0.693
Loin muscle area, cm^2^	14.23	13.38	0.461	0.385
GR, mm	10.00	8.00	0.118	0.166

**Table 5:** Effect of oligosaccharides chelated with organic trace minerals on meat quality indices of mutton sheep.

Items	CON	OTM	SEM	*p* value
pH_45min_	6.43	6.58	0.078	0.188
pH_24h_	5.62	5.82	0.083	0.104
Shear force, kg	6.86	5.41	0.588	0.084
Cooking loss, %	37.74	31.35	2.316	0.192
Drip loss, %	14.98	16.24	4.692	0.858
Muscle chemical composition				
Moisture, %	74.04	77.00	0.859	0.072
Crude protein, % dry matter	81.96^b^	83.27^a^	0.330	0.048
Ether extract, % dry matter	11.34^a^	8.46^b^	0.539	0.020
Organic matter, % dry matter	96.77	96.58	1.052	0.906

**Table 6:** Effect of oligosaccharides chelated with organic trace minerals on amino acid content of meat from mutton sheep.

Items		CON	OTM	SEM	*p* value
EAA	Thr	40.30^b^	77.49^a^	4.746	0.001
	Val	73.36^b^	115.73^a^	8.326	0.003
	Met	32.85	42.70	6.823	0.152
	Ile	40.56	57.93	10.024	0.101
	Leu	78.52^b^	116.50^a^	14.039	0.030
	Phe	39.87	52.68	5.750	0.053
	Trp	10.80	15.63	2.150	0.051
	Lys	129.87^b^	218.22^a^	16.191	0.003
HEAA	His	23.16	34.63	5.610	0.066
	Arg	89.93^b^	164.16^a^	14.001	0.003
NEAA	Gln	262.15	250.83	11.617	0.298
	Glu	27.72	63.58	7.133	0.132
	Gly	83.58^b^	123.44^a^	2.749	<0.001
	Ala	377.51	393.10	26.468	0.511
	Ser	37.22^b^	76.92^a^	4.764	0.001
	Pro	38.38^b^	89.76^a^	3.827	<0.001
	Tyr	32.93^b^	48.42^a^	5.520	0.026
	Cys	0.90^b^	1.30^a^	0.079	0.003
	Asn	19.62	25.17	3.932	0.159
	Asp	12.67^b^	42.83^a^	5.991	0.033
TAA		1451.90^b^	2011.02^a^	133.966	0.004
EAA		446.13^b^	696.88^a^	59.304	0.005
HEAA		113.09^b^	198.79^a^	19.819	0.002
NEAA		892.68^b^	1115.36^a^	61.402	0.034
UAA		572.28	724.05	46.402	0.075
EAA/TAA,%		30.72	34.65	0.012	0.131
EAA/NEAA,%		49.97	62.48	0.037	0.080

^1^Thr, threonine; Val, valine; Met, methionine; Ile, isoleucine; Leu, leucine; Phe, phenylalanine; Trp, tryptophan; Lys, lysine; His, histidine; Arg, argnine; Gln, glutamine; Glu, glutamic acid; Gly, glycine; Ala, alanine; Ser, serine; Pro, proline; Tyr, tyrosine; Cys, cysteine; Asn, asparagine; Asp, aspartic acid; TAA, total amino acid; EAA, essential amino acid; HEAA, semiessential amino acid; NEAA, nonessential amino acid; UAA, umami amino acid (Gly, Ala, Asp, Glu, Phe, Tyr). ^2^Data in the same row with different letters are significantly different (*p* < 0.05), whereas data without letters are not significantly different (*p* > 0.05), and the following tables are the same.

## 4 Discussion

In large-scale animal breeding, trace minerals play a critical role in weight gain and FCR, as well as in body structure, physiological functions, and hormone regulation (22). Previous studies have shown that trace minerals, such as copper and zinc, regulate animal appetite and improve animal feed intake. For example, copper stimulates the hypothalamus to secrete neuropeptide γ (a hormone that stimulates food intake), thus improving the feed intake of pigs (23). Zinc stimulates the regeneration of animal taste bud cells, increases animal appetite, improves intestinal flora, elevates FCR (24), and increases DMI in dairy cows (25). Compared to inorganic trace elements, organic chelated trace elements have the advantages of good palatability, high absorption efficiency, and small antagonism between minerals, which can improve feed intake and ADG of Angus calves before weaning (26). However, some studies have shown that organic trace elements do not exert a regulatory effect on animal feed intake. For example, cows fed lick blocks containing cobalt, iron, selenium, manganese, and zinc have no significant effect on DMI (27). Also, ewes-fed lick blocks containing iron, copper, zinc, manganese, cobalt, and selenium did not show any significant change in feed intake (28). Organic trace minerals have no significant effect on animal feed intake but improve FCR (29). This phenomenon could be attributed to the fact that animal appetite is affected by various factors, such as environment, gastrointestinal content, blood glucose, mental status, and neurological function (30), such that the effect of trace mineral supplementation on an animal’s appetite and feed intake may depend on the factors mentioned above. In this study no significant change was detected in ADG, DMI and FCR of mutton sheep in the OTM group.

The digestive enzyme activities of the gastrointestinal tract reflect the digestive and absorptive ability of nutrients, which has a close correlation with the animal’s production performance. The jejunum is the main organ of nutrient digestion and absorption. It is reported that increasing supplementation of Zn, and Cu and can increase trypsin, lipase amylase activity (31, 32). In this study, the trypsin, lipase, and amylase activities in the jejunum chyme of the OTM group were significantly increased, which indicated that trace mineral supplementation is more efficient. Furthermore, it was expected to improve the digestibility of feed CP, EE, and energy by the micronutrients (33). However, the FCR showed no significant difference, it may be related to ruminant digestion or the consumption of animals, which needs more research to confirm. On the other hand, OTM increased the immunoglobulin levels in the blood but decreased the inflammatory response indicators and the IFN-γ level, which indicated the potential increase of the humoral and innate immunity of the animals (34, 35).

Antibodies (immunoglobulins) are major indicators of the animal’s immune capacity. Immunoglobulins (IgA, IgM, and IgG) are associated with blood diseases, infections, and autoimmune diseases (36). Among these, IgG constitutes the largest proportion and is vital in maintaining the body’s immune barrier (37) and in the early defense of the body. IgA has anti-viral and bacteriostatic immunological activities, is the main effector molecule of humoral and mucosal immunity, and directly participates in immune responses. IgM is a high-performance antibody in the body’s defense and immune process, with bactericidal function and antiviral (38). Previous studies have shown that the addition of zinc glycinate chelate to diets increases the serum IgA, IgM, and IgG levels (39). Supplementation of manganese and selenium in the diets of goats and lambs increases the serum IgA, IgM, and IgG content (40, 41). It was reported that replacing 1/3 of the trace element inorganic salt in the diet with chelated organic trace elements can significantly increase the serum IgG content of piglets (42). IgA, IgM, and IgG were significantly higher in the serum of mutton sheep in the OTM group in this experiment, which was consistent with the previously reported results, indicating that OTM enhances the immune competence and health of the animals. This phenomenon may be related to the fact that organic trace minerals promote the proliferation and differentiation of lymphocytes and increase the number of plasma cells, reducing inflammation and enhancing the immune capacity of the body (43). Additionally, trace minerals, such as manganese, zinc, copper, and selenium enhance humoral and cell-mediated immune responses, maintain oxidative balance in the body, reduce morbidity and treatment costs in cattle, and positively affect breeding (44).

Furthermore, the increase of immunoglobulins in the OTM group may be also relate to the immune stimulation of oligosaccharides (45, 46). Some oligosaccharide (such as mannan) itself is involved in the formation of immunoglobulins (glycoproteins) (47). Therefore, the OTM may provide oligosaccharides for immune synthesis. Blood biochemical indices reflect the nutritional metabolism of mutton sheep (48). The protein content in the serum is closely linked to protein uptake and metabolism, as many proteins in the serum act as molecular carriers of nutrients, hormones, or metals and are extensively involved in a wide range of functions in the organism (49). The TP content is related to the nutritional status blood capacity and stress of animals (50), etc. The lower TP in the CON group indicates poorer health condition of mutton sheep, which increases the proportion of protein dealing with harsh environments or immune depletion and it is not conducive to improving nutrient utilization. On the other hand, ALB in sheep blood is correlated significantly and positively with TP (51). It is a marker of liver function, and higher values of ALB imply improved liver function (52). The liver plays a vital role in the digestion of nutrients, which might explain the improved feed efficiency of the OTM group. Moreover, serum GLB mainly includes IgG, IgA, and IgM. These immunoglobulins regulate the immune system of the animals, and the increased GLB level enhances humoral immune responses against pathogenic viruses and microorganisms (53). Blood TP, ALB, and GLB were significantly increased in the OTM group, indicating that OTM provides an immune-enhancing environment that is beneficial to improving the performance of mutton sheep (54). This may be also related to increased activity and functions of rumen microorganisms by organic selenium (55). This further improves the utilization of ammonia in the rumen, and increases the blood urea nitrogen that can be used to synthetic proteins.

The level of cytokines in serum is a major indicator for evaluating the immune function of the body, i.e., immune regulation, inflammatory response, and neuroendocrine function. Among these, IL-10 regulates the anti-inflammatory cytokines and immune responses (56). Some studies have shown that IL-10 inhibits the production and expression of IL-2, IL-6, and IFN-γ (57). It also promotes B cell proliferation, differentiation, and antibody production while inhibiting the secretion of pro-inflammatory factors, such as IL-6 via macrophages (58). and IL-10 inhibits the synthesis of Th1 cells (59). Since IL-2 and IFN-γ are secreted by Th1 cells (60), IL-10 inhibits the production and expression of pro-inflammatory factors, such as IL-2, IL-6, and IFN-γ. IL-2 is a crucial Pro-inflammatory factor that regulates T cell proliferation and secretion (61). It also stimulates the production of IFN-γ by Th1 cells, which in turn affects the IFN-γ-mediated immune response. Interestingly, IL-6 has a dual role in the animal’s organisms: as a pro-inflammatory factor that judges the inflammatory situation in the organism (62), and as a factor that promotes the production of IgM, IgG, and IgA through mature B cells (63). In this study, the serum levels of IL-10 in mutton sheep were significantly higher, while IL-2, IL-6, and IFN-γ were significantly lower in the OTM group than in the CON group. Thus, we speculated that OTM promotes the proliferation and differentiation of B cells, which was also evident from the content of IgA, IgM, and IgG antibodies in the blood of mutton sheep, while the production of IL-2, IL-6, and IFN-γ was suppressed; this finding was in agreement with the results reported previously and could be related to the modulation of cytokines by certain mineral elements. For example, zinc induces the proliferation of T lymphocytes by inhibiting IL-2 factor (64). and decreases the levels of pro-inflammatory cytokines, such as IL-6 (65). Selenium reduces the IL-2 levels in mouse serum (66). Fe increases the bactericidal and phagocytic capacity of neutrophils, improves the proliferation and differentiation of T and B lymphocytes, and promotes the production of antibodies (67). Copper has a dual effect on cytokines in rat kidney tissues, promoting cell proliferation at low doses and inhibiting proliferative activity at high doses (68). Moreover, the cytokines in animal organisms are affected by a single trace mineral and the combined effects of multiple trace minerals. For example, on the basal diet of high-sugar rats, a mixed solution containing 11 trace minerals (B, V, Cr, Mn, Fe, Co, Cu, Zn, Se, Sr., and Mo) was instilled, which increased the IL-10 but significantly decreased the IL-6 level in rat serum. This phenomenon could be attributed to the fact that supplementation of multiple trace minerals upregulates the expression of IL-10 and downregulates the expression of IL-6, thus reducing the inflammatory response in rats fed a high-sugar diet (69). This, influencing the secretion of cytokines by immune cells is the result of a comprehensive effect of a variety of trace minerals. In this study, OTM increased the IL-10 content in the blood of mutton sheep and decreased the content of IL-2, IL-6, and IFN-γ, suggesting that OTM promotes the proliferation and differentiation of the organism’s B cells. In addition, the synthesis of Th1 cells was inhibited, which might be related to the provision of organic trace minerals and the enhancement of the secretory function of B cells as well as immune cell activity. However, further immunological experiments are needed to substantiate this finding.

Slaughter performance is a major index to measure the economic performance of meat livestock; the items include live weight before slaughter, carcass weight, dressing percentage, and eye muscle area. Studies have shown that supplemental feeding of trace mineral lick bricks significantly improves the dressing percentage and eye muscle area of semi-fine wool sheep in Guizhou and thus, the economic benefits of farming (70). Tibetan sheep supplemented with lick bricks containing manganese, iron, copper, iodine, zinc, selenium, cobalt, and other composite trace mineral nutrients show a significantly increased dressing percentage, net meat rate, eye muscle area, and carcass quality (70, 71). In this trial, the slaughter differences and the body weights of the mutton sheep were similar between the two groups, which was different from previous reports. This might be because the licked bricks in the previous studies were an additional supplement and exhibited an incremental effect whereas in the present trial, the supplementation was rationed according to the nutrient needs of the goats. Typically, the intake of microminerals of the mutton sheep in the two groups was adequate, such that the OTM group did not reflect an incremental effect; however, this finding needs to be confirmed by further investigation. Meat quality reflects the taste and food value of mutton. Interestingly (72), the meat will have a better flavor and color and will be more palatable to the consumer at pH 5.3–5.8 than at pH 6.6–7.0. In the present study, the pH of the longest muscle of the back of the two groups of mutton sheep decreased from 6.4–6.6 at 45 min of slaughter to 5.6–5.8 after 24 h of acid drainage, indicating that the meat of the test goats underwent a desirable maturation process, which is consistent with the results of the previous study. Muscle tenderness is also a critical indicator for evaluating the texture of meat, which is measured by shear force: the lower the shear force, the finer the muscle fibers, the more tender the meat, and the better the texture. A study on whether it is acceptable for people to pay a premium for buying more tender meat showed that meat can be categorized as very soft (2.27–3.58 kg), medium soft (4.08–5.40 kg), and mildly soft (5.90–7.21 kg) based on the shear force profile of the meat (73), with the more tender meat as preferable. In this study the shear force of 5.41 kg in the OTM group was classified as medium soft meat and 6.86 kg in the CON group was classified as light soft meat. In comparison, the lamb meat in the OTM group was softer and easier to chew, which was in line with people’s tendency to choose tenderness. In addition, drip loss and cooking loss are indicators of muscle tethering force, which affect the color, flavor, tenderness, and nutritional value of meat. Meat with high hydraulics tends to be juicy, tender, and dry on the surface. On the other hand, meat with low hydraulics suffers from surface water exudation, loss of soluble nutrients, and flavor changes, the muscle becomes dry and tough, and the quality of the meat decreases. Also, high cooking losses make the meat tough and intolerant to chewing after cooking, which reduces the flavor (74). Herein, we did not detect any significant difference between drip loss and cooking loss between the two groups of lamb, indicating that OTM does not alter the cooking quality.

In terms of ether extract, the breed of sheep exerts a significant influence. Merino sheep have a lower ether extract content and a high protein content, with nine amino acids higher than that of the small-tailed Han sheep (75). In addition, nutrition affects the deposition of ether extract. Some studies have shown that supplementation with Cu (40 mg/kg DM) and Se (2 mg/kg DM) reduces cholesterol deposition in the longest muscle of the back of Brangus cattle, possibly due to the alteration of the ratio between reduced and oxidized glutathione, which in turn affects lipid metabolism of the cattle (76). Adding Cu to the diet of Angus beef cattle reduces the ether extract content of the longissimus muscle of the back (77). Selenium regulates lipid metabolism and ether extract accumulation in muscle by affecting thyroid hormones (78). High zinc increases the activity of rat liver cells and upregulates fat synthesis genes, which in turn promotes lipid metabolism in dirty cells (79). In this experiment study, the content of EE in meat was significantly increased, while the content of CP was significantly increased, indicating that the addition of organic trace minerals changed the composition of meat. However, the raised protein content did not increase the shear force, which might be related to factors such as muscle fiber diameter or connective tissue content; nonetheless, these findings need to be confirmed by further studies.

The CP content of meat is the basis for measuring meat quality, and its level is related to the amino acid content and composition of the meat. Based on the nutritional aspect the amino acid content of the meat determines the flavor and nutritional value of meat and a high content of essential amino acids can satisfy human nutritional requirements (80). The analyses revealed that the content of all amino acids was higher in the OTM group than in the CON group. Among these, the essential amino acids Thr, Val, Leu, and Lys and the total number of EAA were significantly higher in the OTM group than in the CON group, which increased the relative protein content in the meat. Therefore, the ability of OTM to promote amino acid synthesis in muscle was superior to that of inorganic trace minerals. This phenomenon may be related to the high absorption and utilization of organic minerals. Previous studies have shown that the addition of yeast selenium to the diet promotes the synthesis of selenate amino acids by rumen bacteria, which in turn, increases the amino acid content of goat meat (81).

Regarding the balance of amino acids and ideal protein, FAO/WTO suggested that the ratio of EAA/TAA is 40%, and EAA/NEAA >60% is considered high-quality protein. In the present study, the EAA/TAA in the CON and OTM groups of lamb was 30.72% and 34.65%, respectively, wherein the ratio of the OTM group was 3.93% higher than that of the CON group and almost 40%. On the other hand, the ratios of EAA/NEAA in mutton of both CON and OTM groups were higher than the standard 60%; the OTM group was 12.51% higher than the CON group and was above the standard. Therefore, it could be deduced that the protein and amino acid quality of the OTM group was better and the nutritional value was richer than that of the CON group. Furthermore, flavor and taste are subjective sensations. Some amino acids are either flavor substances or precursors for the formation of flavor substances that can be used to evaluate the flavor of the meat. Previous studies have shown that Glu and Asp are the characteristic amino acids for fresh flavor, Ser, Ala, Gly, Thr, and Pro are responsible for sweetness, and Leu and Phe form the bitterness (82–84). Other studies have shown that trace mineral supplementation modulates the content of umami amino acids (UAA). For example, yeast Se supplementation reduced the dissipation of fresh flavor in lamb meat and increased the content of five umami amino acids (UAA; Ala, Asp, Glu, Phe, and Tyr) in lamb meat (85). Taken together, the current results showed that OTM increases the content of various fresh amino acids and improves the flavor of lamb meat.

## 5 Conclusion

Oligosaccharide-chelated organic trace minerals supplemented in the diet of mutton sheep could increase the jejunal digestive enzyme activity, the immune level in the blood, and improve the meat quality, but had no significant effect on growth performance, feed conversion ratio and slaughter parameters.

## Data availability statement

The original contributions presented in the study are included in the article/supplementary material, further inquiries can be directed to the corresponding author.

## Ethics statement

The animal studies were approved by Laboratory Animal Ethics Committee of Inner Mongolia Minzu University College of Animal Science and Technology. The studies were conducted in accordance with the local legislation and institutional requirements. Written informed consent was obtained from the owners for the participation of their animals in this study.

## Author contributions

RZ: Writing – original draft, Writing – review & editing. MW: Writing – original draft, Writing – review & editing. JZ: Writing – original draft. ZY: Writing – original draft, Supervision. MX: Writing – original draft, Data curation. LD: Writing – original draft. MB: Writing – original draft. JJ: Writing – original draft. CD: Writing – original draft. YZ: Writing – original draft. HB: Writing – original draft.
